# Adults with adhd symptoms express a better inhibitory capacity when the perceptual load is higher

**DOI:** 10.1192/j.eurpsy.2021.1631

**Published:** 2021-08-13

**Authors:** L.R. Carreiro, W. Machado-Pinheiro, A. Afonso Junior

**Affiliations:** 1 Developmental Disorders Graduate Program, Mackenzie Presbyterian University, São Paulo, Brazil; 2 Departamento De Ciências Da Natureza, Universidade Federal Fluminense, Rio das Ostras, Brazil

**Keywords:** Stop-signal reaction time, ADHD, Inhibitory process

## Abstract

**Introduction:**

ADHD is associated with impairments in different inhibitory functions, including suppression of an already initiated response and inhibition of distracting information. This work used a protocol that combines the Stroop-matched and stop-signal tasks to examine the association between the frequency of ADHD symptoms and different inhibitory abilities in a young adult.

**Objectives:**

To investigate how the symptoms of inattention and hyperactivity / impulsivity are associated with three forms of inhibition evaluated by the Stroop-matched / stop-signal task: inhibiting an automatic response, controlling interference and canceling a response.

**Methods:**

38 participants (33 women; mean age = 23.3; SD = 5.17) completed Adult ADHD Self-Report Scale (ASRS) assessing ADHD symptoms before performing the task. Reaction times, accuracy and stop-signal reaction time (SSRT; the latency of the inhibitory process of response cancellation) were calculated for each task condition.

**Results:**

There was a significant correlation of ADHD symptoms and SSRT in the condition with the higher perceptual load (i.e., a greater number of colors presented in the same test). This correlation was negative (r = - .36, p <.05), which indicates that participants with higher ADHD symptoms frequency had more efficient inhibitory processes in this condition.

**Conclusions:**

(1) the perceptual load of the task influences the cancellation of responses; (2) individuals with higher frequency of ADHD symptoms may have a better inhibitory capacity when the perceptual load is high, possibly reflecting a lower availability of attentional resources to process distracting information.

**Disclosure:**

No significant relationships.

